# Supplementary material for: Phylogenetic Analysis and Development of Molecular Tool for Detection of Diaporthe citri Causing Melanose Disease of Citrus
Source: Plants (Basel). 2020 Mar 4;9(3):329. doi: 10.3390/plants9030329 (PMC7154919; doi:10.3390/plants9030329)
Supplement: Supplementary file 1 [file plants-09-00329-s001.pdf]

plants

MDPI

Article

Phylogenetic Analysis and Development of Molecular Tool for Detection of *Diaporthe citri* Causing Melanose Disease of Citrus

Chingchai Chaisiri [1,2], Xiang-Yu Liu [1,2], Yang Lin [1], Jiang-Bo Li [3], Bin Xiong [3] and Chao-Xi Luo [1,2,]*

1 Key Lab of Horticultural Plant Biology, Ministry of Education, Huazhong Agricultural University, Wuhan 430070, China; chaisiri.ch@gmail.com2425498425@qq.com (C.C.); tj1009lxy@qq.com (X.-Y.L.); yanglin@mail.hzau.edu.cn (Y.L.)

2 Department of Plant Pathology, College of Plant Science & Technology, and Key Lab of Crop Disease Monitoring & Safety Control in Hubei Province, Huazhong Agricultural University, Wuhan 430070, China

3 Nanfeng Citrus Research Institute, Nanfeng 344500, China; fzgyjljb@163.com283270130@qq.com (J.-B.L.); fzsgyj@163.com1312314614@qq.com (B.X.)

* Correspondence: cxluo@mail.hzau.edu.cn

Received: 16 February 2020; Accepted: 27 February 2020; Published: 4 March 2020date

Abstract: Melanose disease caused by *Diaporthe citri* is considered as one of the most important and destructive diseases of citrus worldwide. In this study, isolates from melanose samples were obtained and analyzed. Firstly, the internal transcribed spacer (ITS) sequences were used to measure *Diaporthe*-like boundary species. Then, a subset of thirty-eight representatives were selected to perform the phylogenetic analysis with combined sequences of ITS, beta-tubulin gene (*TUB*)*TUB*, translation elongation factor 1-α gene (*TEF*)*TEF*, calmodulin gene (*CAL*), and histone-3 gene (*HIS*)*CAL*, and *HIS*. . As a result, these representative isolates were identified belonging to *D. citri*, *D. citriasiana*, *D. discoidispora*, *D. eres*, *D. sojae*, and *D. unshiuensis*. Among these species, the *D. citri* was the predominant species that could be isolated at highest rate from different melanose diseased tissues. The morphological characteristics of representative isolates of *D. citri* were investigated on different media. Finally, a molecular tool based on the novel species-specific primer pair TUBDcitri-F1/TUBD-R1, which was designed from highly conserved region of *TUB* gene, was developed to detect *D. citri* efficiently. A polymerase chain reaction (PCR) amplicon of 217 bp could be specifically amplified with the developed molecular tool. The sensitivity of the novel species-specific detection was upon to 10 pg of *D. citri* genomic DNA in a reaction. Therefore, the *D. citri* could be unequivocally identified from closely related *Diaporthe* species by using this simple PCR approach.

Keywords: *Citrus*; *Diaporthe citri*; geographical distribution; molecular diagnostics; multi-locus phylogenetics

1. Introduction

Citrus and their allied genera (including *Eremocitrus*, *Fortunella*, *Microcitrus*, and *Poncirus*) are widely distributed worldwide, among them, the most popular cultivars belong to the Aurantioideae subfamily of the Rutaceae family. Allegedly, the citrus was originally cultivated in Himalayas 4000 years ago [1]. Nowadays, *Citrus* is one of the most widely cultivated fruit crops with a planting area of 2.5 million ha and production of more than 38 million tons per year in China [2]. The popular citrus cultivars in China include *Citrus reticulata* (mandarin), *Citrus sinensis* (sweet orange), *Citrus grandis* or *Citrus maxima* (pumelo), and *Citrus paradisi* (grapefruit) [3].

Commented [M1]: Please carefully check the accuracy of names and affiliations.

Commented [ชไ2]: confirmed

Commented [M3]: QQ mailbox is not allowed. Please provide the institute email address.

Commented [LS4]: Please ensure that all unfamiliar abbreviations are defined the first time they're used, in both the abstract and main text, as well as any figure subtitles/captions of an article.

Formatted: Font: 8 pt

The *Diaporthe* genus fungi are well-known as saprobic-, endophytic-, and pathogenic-plant parasites on economically significant plant cultivars [4–8]. One host species can be affected by many different *Diaporthe* species, whereas one *Diaporthe* species can infect many hosts species [9–13]. Accurate identification of *Diaporthe* species is very important for controlling the diseases caused by these fungi and making effective quarantine strategies [14–17].

The *Diaporthe citri* (syn. *Phomopsis citri*) has a wide spectrum on several citrus species including mandarin, sweet orange, pumelo, grapefruit, and lemons [18]. A potential damage referred multiple symptoms e.g., wood canker, twig blight, brunch dieback, gummosis, stem-end rot, and melanose [18–24]. The melanose, one of the most serious citrus diseases caused by *D. citri* was firstly reported on citrus fruits in Florida [25]. In 1912, Fawcett [26] reported that stem-end rot was caused by *Phomopsis citri*, while Floyd and Stevens [27] provided the evidence that stem-end rot and melanose disease were infected by the same fungus. In 1914, a fungus *Diaporthe citrincola* was firstly collected and described from twigs of *Citrus nobilis* [28]. In 1917, *Phomopsis caribaea* was reported on twigs of grapefruit in Isle of Pines, Cuba [29]. In early studies, *D. citri* was reported in several names including *Diaporthe medusaea* [30], *Phomopsis californica* [31], and *Phoma cytosporella* [28]. In 1928, Bach and Wolf [32] fulfilled Koch's postulates for *D. citri* infection on citrus. Pathogenicity test demonstrated that both conidiospore of *P. citri* and ascospore of *D. citri* could produce leaf melanose symptoms [33].

Traditional molecular barcoding for fungal species discrimination based on nuclear ribosomal internal transcribed spacer regions (ITS) is frequently used for the identification of *Diaporthe* genus [7,34–36]. The molecular phylogeny based on the combination of multi-locus DNA sequences showed better identification of *Diaporthe* species [6,20,37–40]. The combination of translation elongation factor 1-α gene (*TEF*), beta-tubulin gene (*TUB*), calmodulin gene (*CAL*), and histone-3 gene (*HIS*) showed good resolution for *Diaporthe* species discrimination [7,38,41]. Generally, molecular marker was used to detect *Diaporthe* species, and many species-specific primers were designed based on conserved ITS region such as in *Diaporthe phaseolorum* and *Diaporthe longicolla* from soybean [42], *Diaporthe azadirachtae* from neem [43,44], *Diaporthe sclerotioides* from plants and soils [45]. Also, a molecular tool based on *TEF* gene was developed to detect *Diaporthe azadirachtae* from neem [46]. However, these methods are hard to distinguish *D. citri* and its closely related species because only limited informative variations could be found in ~~t~~both the ITS region and *TEF* gene, ~~that~~thus, it is hard to design specific primers based on these sequences to distinguish *D. citri* from other *Diaporthe* species.

The aims of this study was to: (i) to define the species discrimination of *D. citri* based on phylogenetic analyses and (ii) to develop a molecular tool to simply detect *D. citri* from multiple *Diaporthe* species on citrus plants.

Formatted: Font: Italic

Commented [LS5]: Please revise, I'm not sure how to edit your intended meaning.

Formatted: Font: Italic

2. Results

2.1. Isolation of Diaporthe Species

Totally 140 isolates were obtained and 38 representative isolates from different tissues, i.e., leaves, fruits, and twigs were selected for further study (Table 1; Figure 1). The identification based on ITS sequence analysis showed that all these isolates belong to *Diaporthe* species (Supplementary Figure S1).

Table 1. Collection details and GenBank accession numbers of isolates included in this study.

***Diaporthe* Species**	**Isolate Number**	**Plant Host**	**Tissue**	**Locality**	**GenBank Accession Numbers** [1]				
					**ITS**	***TUB***	***TEF***	***CAL***	***HIS***
D. citri	NFFF-1-2	*Citrus reticulata* cv. *Nanfengmiju*	fruit	China: Jiangxi: Nanfeng	MN816394	MN894454	MN894415	MN894355	MN894380
	NFFF-1-4	*Citrus reticulata* cv. *Nanfengmiju*	fruit	China: Jiangxi: Nanfeng	MN816395	MN894455	MN894416	MN894356	MN894381
	NFFF-2-5	*Citrus reticulata* cv. *Nanfengmiju*	fruit	China: Jiangxi: Nanfeng	MN816396	MN894456	MN894417	MN894357	MN894382
	NFFL-1-13	*Citrus reticulata* cv. *Nanfengmiju*	leaf	China: Jiangxi: Nanfeng	MN816397	MN894457	MN894418	MN894358	–
	NFFL-1-8	*Citrus reticulata* cv. *Nanfengmiju*	leaf	China: Jiangxi: Nanfeng	MN816398	MN894458	MN894419	MN894359	–
	NFHF-8-4	*Citrus reticulata* cv. *Nanfengmiju*	fruit	China: Jiangxi: Nanfeng	MN816399	MN894459	MN894420	MN894360	–
	NFHL-7-11	*Citrus reticulata* cv. *Nanfengmiju*	leaf	China: Jiangxi: Nanfeng	MN816400	MN894460	MN894421	MN894361	MN894383
	NKDL-2-17	*Citrus sinensis*	leaf	China: Jiangxi: Nankang	MN816401	MN894461	MN894422	MN894362	MN894384
	NKCL-6-12	*Citrus sinensis*	leaf	China: Jiangxi: Nankang	MN816402	MN894462	MN894423	MN894363	MN894385
	NKCT-6-24	*Citrus sinensis*	twig	China: Jiangxi: Nankang	MN816403	MN894463	MN894424	MN894364	MN894386
D. citriasiana	XFAL-1-1	*Citrus sinensis*	leaf	China: Jiangxi: Xinfeng	MN816404	MN894464	MN894425	–	MN894387
	NFFL-2-41	*Citrus reticulata* cv. *Nanfengmiju*	leaf	China: Jiangxi: Nanfeng	MN816405	MN894465	MN894426	–	MN894388
	XFKL-15-2	*Citrus sinensis*	leaf	China: Jiangxi: Xinfeng	MN816406	MN894466	MN894427	–	MN894389
D. discoidispora	NFJF-1-1	*Citrus reticulata* cv. *Nanfengmiju*	fruit	China: Jiangxi: Nanfeng	MN816407	MN894467	MN894428	–	MN894390
	NKDL-1-2	*Citrus sinensis*	leaf	China: Jiangxi: Nankang	MN816408	MN894468	MN894429	–	MN894391
	NKDL-2-3	*Citrus sinensis*	leaf	China: Jiangxi: Nankang	MN816409	MN894469	MN894430	–	MN894392
	NKDL-1-6	*Citrus sinensis*	leaf	China: Jiangxi: Nankang	MN816410	MN894470	MN894431	–	MN894393
	NFFL-3-46	*Citrus reticulata* cv. *Nanfengmiju*	leaf	China: Jiangxi: Nanfeng	MN816411	MN894471	MN894432	–	MN894394
D. eres	NFFL-1-25	*Citrus reticulata* cv. *Nanfengmiju*	leaf	China: Jiangxi: Nanfeng	MN816412	MN894472	MN894433	–	MN894395
	NFFL-1-36	*Citrus reticulata* cv. *Nanfengmiju*	leaf	China: Jiangxi: Nanfeng	MN816413	MN894473	MN894434	MN894365	MN894396
	NFFL-2-17	*Citrus reticulata* cv. *Nanfengmiju*	leaf	China: Jiangxi: Nanfeng	MN816414	MN894474	MN894435	–	MN894397
	NFFL-2-8	*Citrus reticulata* cv. *Nanfengmiju*	leaf	China: Jiangxi: Nanfeng	MN816415	MN894475	MN894436	MN894366	MN894398
	NFFL-3-1	*Citrus reticulata* cv. *Nanfengmiju*	leaf	China: Jiangxi: Nanfeng	MN816416	MN894476	MN894437	MN894367	MN894399
	NFFL-4-5	*Citrus reticulata* cv. *Nanfengmiju*	leaf	China: Jiangxi: Nanfeng	MN816417	MN894477	MN894438	MN894368	MN894400
	NFFT-3-3	*Citrus reticulata* cv. *Nanfengmiju*	twig	China: Jiangxi: Nanfeng	MN816418	MN894478	MN894439	–	MN894401
	NFFT-3-8	*Citrus reticulata* cv. *Nanfengmiju*	twig	China: Jiangxi: Nanfeng	MN816419	MN894479	MN894440	MN894369	MN894402
	NFIF-1-1	*Citrus reticulata* cv. *Nanfengmiju*	fruit	China: Jiangxi: Nanfeng	MN816420	MN894480	MN894441	MN894370	MN894403
	NFIF-1-7	*Citrus reticulata* cv. *Nanfengmiju*	fruit	China: Jiangxi: Nanfeng	MN816421	MN894481	MN894442	–	MN894404
D. sojae	NFGL-1-5	*Citrus reticulata* cv. *Nanfengmiju*	leaf	China: Jiangxi: Nanfeng	MN816422	MN894482	MN894443	MN894371	MN894405
	NFIT-3-13	*Citrus reticulata* cv. *Nanfengmiju*	twig	China: Jiangxi: Nanfeng	MN816423	MN894483	MN894444	MN894372	MN894406
	NFIF-1-10	*Citrus reticulata* cv. *Nanfengmiju*	fruit	China: Jiangxi: Nanfeng	MN816424	MN894484	MN894445	MN894373	MN894407
	NFFL-1-27	*Citrus reticulata* cv. *Nanfengmiju*	leaf	China: Jiangxi: Nanfeng	MN816425	MN894485	MN894446	MN894374	MN894408
	NFGL-1-7	*Citrus reticulata* cv. *Nanfengmiju*	leaf	China: Jiangxi: Nanfeng	MN816426	MN894486	MN894447	MN894375	MN894409
D. unshiuensis	NFIF-1-6	*Citrus reticulata* cv. *Nanfengmiju*	fruit	China: Jiangxi: Nanfeng	MN816427	MN894487	MN894448	MN894376	–
	NFFT-4-5	*Citrus reticulata* cv. *Nanfengmiju*	twig	China: Jiangxi: Nanfeng	MN816428	MN894488	MN894449	–	MN894410
	NKCT-6-4	*Citrus sinensis*	twig	China: Jiangxi: Nankang	MN816429	MN894489	MN894450	–	MN894411
	NKCL-6-15	*Citrus sinensis*	leaf	China: Jiangxi: Nankang	MN816430	MN894490	MN894451	MN894377	MN894412
	NKCT-6-20	*Citrus sinensis*	twig	China: Jiangxi: Nankang	MN816431	MN894491	MN894452	MN894378	MN894413

[1] ITS = nuclear ribosomal internal transcribed spacer regions; *TUB* = beta-tubulin gene; *TEF* = translation elongation factor 1-α gene; *HIS* = histone-3 gene; and *CAL* = calmodulin gene.

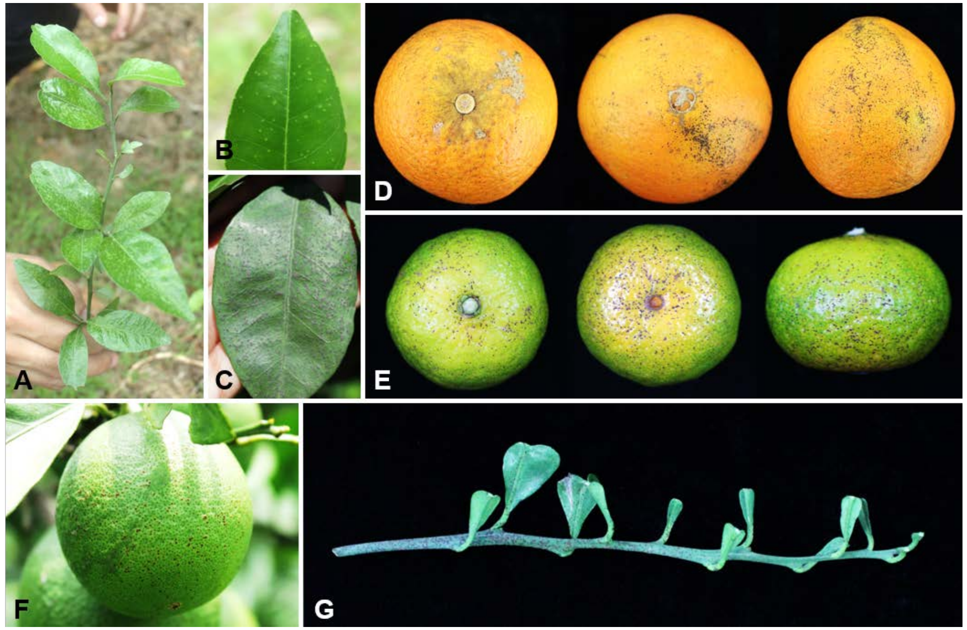

Figure 1. Symptoms of citrus melanose caused by *Diaporthe* species. (**A**,**B**) Typical symptoms on young leaf of *Citrus reticulata* cv. *Nanfengmiju*. (**C**) Typical symptoms on old leaf of *C. sinensis*. (**D**,**E**) Typical symptoms on mature fruits of *C. sinensis* and *C. reticulata* cv. *Nanfengmiju*, respectively. (**F**) Typical symptoms on young fruit of *C. sinensis*. (**G**) Twig typical symptoms of *C. sinensis*.

2.2. Geographic Distribution of *D. citri*

According to the Systematic Mycology and Microbiology Laboratory, ARS, USDA (SMML database), *D. citri* has been recorded on citrus cultivars and their allied genera worldwide. The *D. citri* is a dominant species in *Diaporthe* genus, which occurs widely in citrus-growing countries, e.g., China, Philippines, Japan, Korea, Thailand, Myanmar, Cambodia, Fiji, Mauritius, USA, Mexico, Haiti, Cuba, Dominican, Panama, Puerto Rico, Venezuela, Trinidad and Tobago, Brazil, Cyprus, Portugal (Azores Islands), New Zealand, Niue, Samoa, Tonga, Cook Islands, Cote d'Ivoire, and Zimbabwe. The detailed citrus host and their allied genera of *Diaporthe* spp., are shown in Figure 2 and Supplementary Table S1.

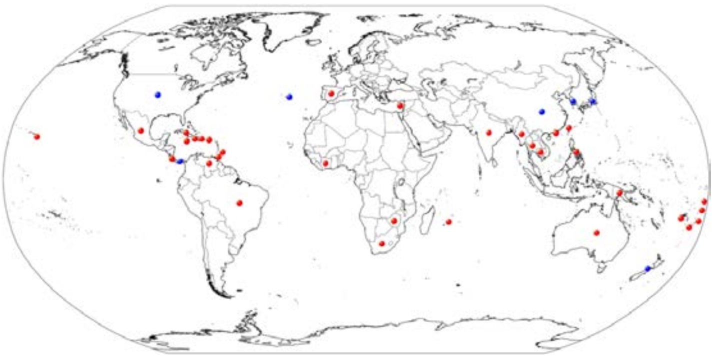

Figure 2. A global geographic distribution of *D. citri* associated with *Citrus*-host plant and available on SMML database. Blue colored dots indicate the availability of the accession numbers in the NCBI database, while red colored dots indicate the non-availability.

2.3. Phylogenetic Analysis of Diaporthe Species

Totally 3183 base pairs (bp) of combined DNA sequences were obtained for phylogenetic analysis, including 645 bp ITS sequence (1–645), 472 bp *TEF* gene sequence (650–1121), 893 bp *TUB* gene sequence (1126–2018), 617 bp *CAL* gene sequence (2023–2639), and 540 bp *HIS* gene sequence (2644–3183). Combined data set consisted of 129 taxa including the outgroup species of *Diaporthella corylina* (CBS 121124). Six phylogenetic trees were constructed corresponding to each single-locus analysis of ITS, *TEF*, *TUB*, *CAL*, *HIS*, and combined data of five loci (Figures 3, Supplementary Figures S1 and S2). The combined data set comprised 56.58% (1801 bp) invariable characters, 31.32% (997 bp) phylogenetically informative characters and 12.10% (385 bp) uninformative variable characters. Each of single locus has the following invariable characters (ITS = 414, *TEF* = 186, *TUB* = 523, *CAL* = 310, and *HIS* = 352), phylogenetically informative characters (ITS = 123, *TEF* = 230, *TUB* = 263, *CAL* = 238, and *HIS* = 143) and uninformative variable characters (ITS = 108, *TEF* = 56, *TUB* = 107, *CAL* = 69, and *HIS* = 45). A comparison of alignment properties in parsimony analyses of gene/loci and nucleotide substitution models used in phylogenetic analyses are provided in Table 2. BI tree constructed with combined five-loci data was presented with annotations for isolate number, plant host, and locality. MP tree was similar to the BI tree, therefore only BI tree was shown. *D. citri* was dominant species and occurred on citrus hosts in countries including China, Japan, Korea, New Zealand, Portugal, and USA. *D. citriasiana* and *D. discoidispora* w~~as~~ere found on citrus plants only. However, *D. eres*, *D. sojae*, and *D. unshiuensis* were found on host plants from multiple genera. Seven isolates obtained in this study clustered in the same group with three isolates from previously known as *D. infertilis* including ex-type strain (CBS 230.52) and several isolates known as *D. citri*, this group should be the *D. infertilis* (Figures 3, S1 and S2). Based on the similar phylogenetic analysis, all of the 140 isolates were identified (Figure S3). Results showed that *D. citri* was the predominant species which accounted for 44.3%, following the species of *D. eres*, *D. unshiuensis*, *D. sojae*, *D.*

Commented [M6]: We changed hyphen into endash. Please confirm this revision.

discoidispora and *D. citriasiana*, which accounted for 11.4%, 10%, 9.3%, 6.4%, and 3.6%, respectively. There were still 15% isolates that could not be identified to the species level (Supplementary Figure S3).

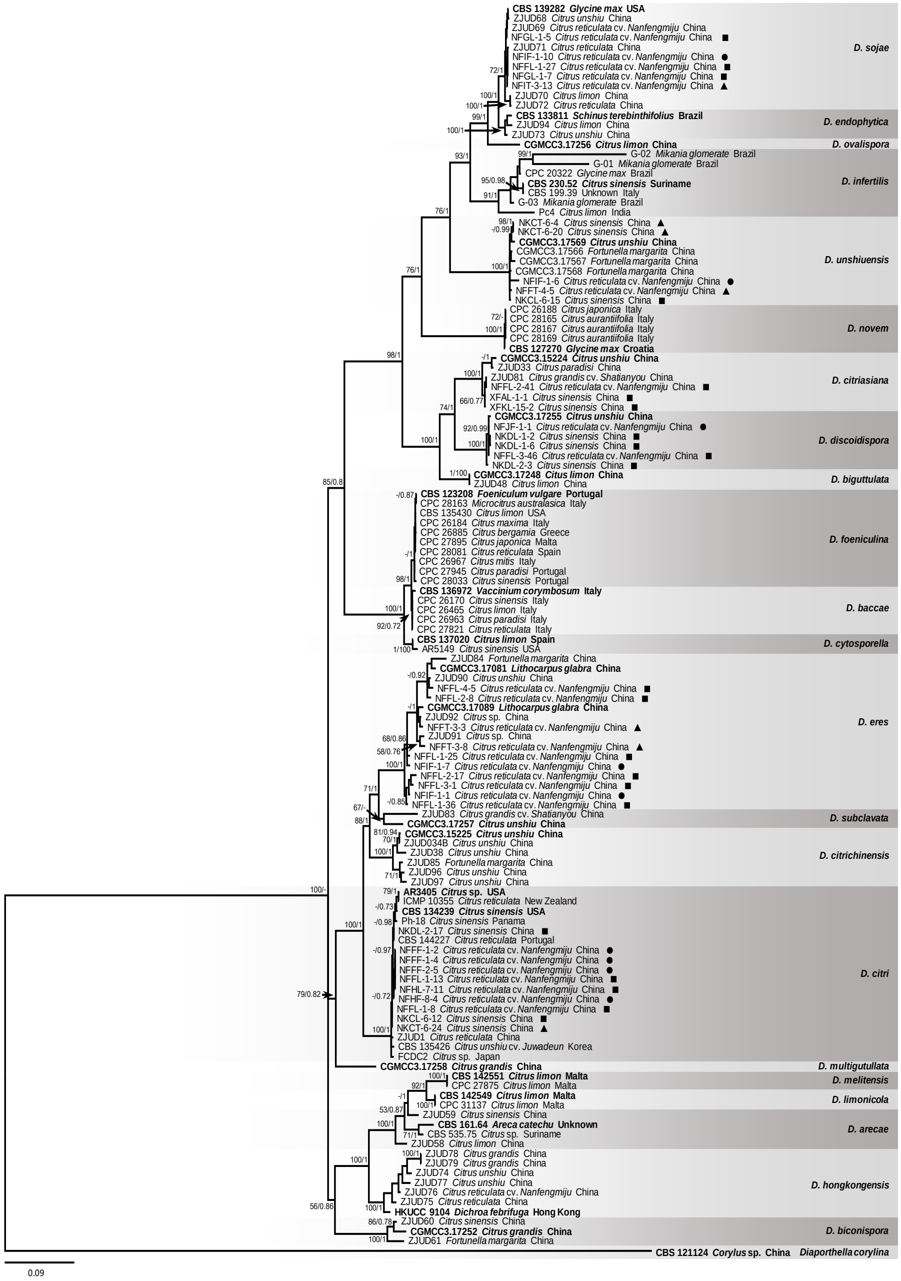

Figure 3. The Bayesian inference consensus tree resulting from a combined data set of ITS, *TUB*, *TEF*, *CAL*, and *HIS* sequences. MP bootstrap support values (equal to or > 50%) and Bayesian posterior probability values (equal to or > 0.70) are indicated at the typological nodes. Ex-type, ex-isotype, and ex-epitype strains are indicated in **bold**. The tree was rooted to *Diaporthella corylina* (CBS 121124). Squares indicate isolates from leaves, circles indicate isolates from fruits, and triangles indicate isolates from twigs. The scale bar represents the expected number of nucleotide substitutions per site.

Table 2. Comparison of alignment properties in parsimony analyses of gene/locus and nucleotide substitution models used in phylogenetic analyses.

Gene/Locus	ITS	*TEF*	*TUB*	*CAL*	*HIS*	Combined
No. of taxa	129	124	124	68	112	129
Aligned length (with gaps)	645	472	893	617	540	3183
Invariable characters (%)	414 (64.19)	186 (39.41)	523 (58.57)	310 (50.24)	352 (65.19)	1801 (56.58)
Phylogenetically informative characters (%)	123 (19.07)	230 (48.73)	263 (29.45)	238 (38.57)	143 (26.48)	997 (31.32)
Uninformative variable characters (%)	108 (16.74)	56 (11.86)	107 (11.98)	69 (11.18)	45 (8.33)	385 (12.10)
Tree length (TL)	670	856	745	554	538	3,654
Consistency index (CI)	0.506	0.575	0.686	0.773	0.55	0.565
Retention index (RI)	0.901	0.948	0.94	0.952	0.926	0.921
Rescaled consistency index (RC)	0.456	0.545	0.645	0.735	0.509	0.521
Homoplasy index (ID)	0.494	0.425	0.314	0.227	0.45	0.435
Nucleotide substitution model	GTR + I + G	GTR + I + G	HKY + G	GTR + G	GTR + I + G	GTR + I + G

2.4. Morphological Characterization of D. citri

For *Diaporthe* species, morphological factors such as colony appearance on different media, conidiomata, conidia shape and size are important to identify and understand a specific species. Therefore, morphological observation was performed on different media. Colonies on PDA grew slowly with 0.3–1.0 mm/day in the dark at 25 °C, they were white, flat or effuse alternate to low convex; reverse mottled buff with irregular dark patches. On CMA and OMA media, sparse to moderate mycelia covered the entire plate after 10 days with numerous scattered pale mouse grey patches. Conidiomata sporulating on PDA were scattered or aggregated, black-deeply embedded in medium, becoming erumpent at maturity. Conidiomata were sub-globose and/or variable in shape and up to 200 μm diam in size with an elongated black neck. Conidial mass was initially hyaline to yellowish, becoming white to cream conidial droplets exuding from central ostioles after 25 days in light at 25 °C. Alpha conidia were aseptate, hyaline, smooth, ovate to ellipsoidal, mostly bi-guttulate, apex bluntly rounded, base sub-truncate, (5.7–) 7–9.2 (–10.1) × (1.7–) 2.1–3.1 (–3.6) μm ($\bar{x}$ ± SD = 8.1 ± 1.1 × 2.6 ± 0.5). Beta conidia were aseptate, flexuous, flexible to slightly curved or hamate, smooth, hyaline, apex acutely rounded, base truncate, (11.7–) 15.7–27.7 (–33) × (0.4–) 0.6–1.2 (–1.6) μm ($\bar{x}$ ± SD = 21.7 ± 6 × 0.9 ± 0.3). Gamma conidia were not observed (Figure 4).

Commented [M7]: We changed hyphen into endash. Please confirm this revision.

Commented [M8]: We changed hyphen into minus. Please confirm this revision.

Commented [M9]: We changed hyphen into endash. Please confirm this revision.

Commented [M10]: We changed hyphen into minus. Please confirm this revision.

Commented [M11]: We changed hyphen into minus. Please confirm this revision.

Commented [M12]: We changed hyphen into endash. Please confirm this revision.

Commented [M13]: We changed hyphen into minus. Please confirm this revision.

Commented [M14]: We changed hyphen into minus. Please confirm this revision.

Commented [M15]: We changed hyphen into endash. Please confirm this revision.

Commented [M16]: We changed hyphen into minus. Please confirm this revision.

Commented [M17]: We changed hyphen into minus. Please confirm this revision.

Commented [M18]: We changed hyphen into endash. Please confirm this revision.

Commented [M19]: We changed hyphen into minus. Please confirm this revision.

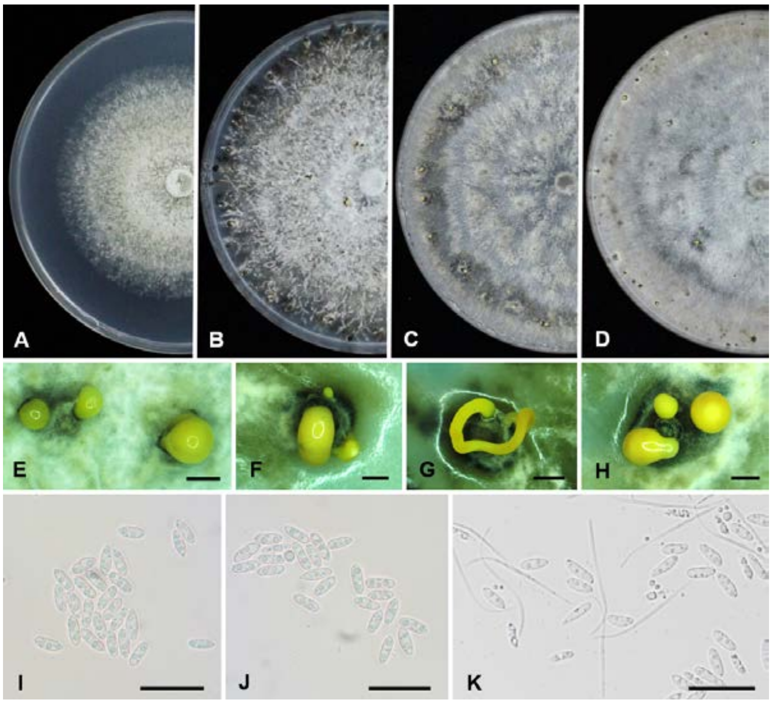

Figure 4. The morphology and cultural characteristics of *D. citri* isolate NFHF-8-4. (**A**,**B**) culture on PDA medium after 7 and 30 days, respectively. (**C**,**D**) colony morphology after 30 days on CMA and OMA media, respectively. (**E**–**H**) mucilaginous drops or tendrils of conidia on PDA. (**I**,**J**) alpha conidia. (**K**) alpha- and beta conidia. Scale bar, **E**–**H** = 200 μm; **I**–**K** = 25 μm.

2.5. Specificity and Sensitivity of PCR Method for Detection of D. citri

As mentioned above, sequences of five loci were obtained for phylogenetic analysis (~~Table 4~~, Supplementary Figures S1 and S2), among them, *TUB* showed the best capability of *D. citri* distinguishing different from other *Diaporthe* species (Figure 5). Therefore, *TUB* gene was chosen for designing the species-specific primers by matching the forward primer in the varied region and the reverse primer in the conserved region of *TUB* gene (Figure 5). As the PCR reaction is performed with the commercial PCR amplification mixture, only the annealing temperature is optimized. Results showed that consistent amplification could be obtained at the annealing temperature from 50 to 60 °C for the species-specific primer pair as shown in Figure 6. Thus, 55 °C was considered as the optimized annealing temperature and used in the following experiments. For the specificity evaluation, the specific primer set TUBDcitri-F1/TUBD-R1 amplified a single product of 217 bp only from the *D. citri* isolates. The 217 bp amplicon was not observed in other five *Diaporthe* species (*D. citriasiana*, *D. discoidispora*, *D. eres*, *D. sojae*, and *D. unshiuensis*), indicating that the method has good specificity for *D. citri* (Figures 7A and S4). The sensitivity was evaluated by using a serial dilution of genomic DNA (gDNA) as templates, results showed that it could amplified the 217 bp fragment from 10 pg of isolate NFHF-8-4 gDNA in 20 μL reaction mixture, indicating very high sensitivity (Figure 7B).

Commented [M20]: We changed hyphen into endash. Please confirm this revision.

Commented [M21]: Please sort the table in the order mentioned.

Commented [ซไ22]: The table 4 also shown in 4.5. Phylogenetic analyses of *Diaporthe* species, and order of table shoud be right now

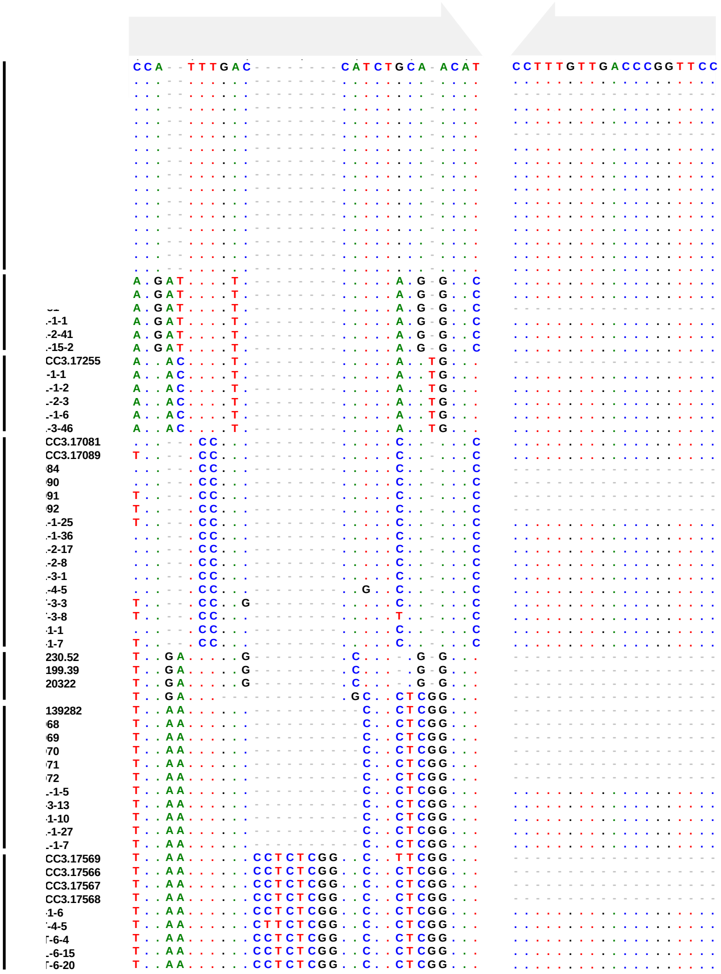

Figure 5. A novel primer pair TUBDcitri-F1 and TUBD-R1 was designed based on the alignment of the partial *TUB* gene (from 5′ to 3′) of *Diaporthe* species including *D. citri*, *D. citriasiana*, *D. discoidispora*, *D. eres*, *D. infertilis*, *D. sojae*, and *D. unshiuensis*. Dashes (−) and dots (.) indicate the gaps and identical nucleotides in the sequences, respectively.

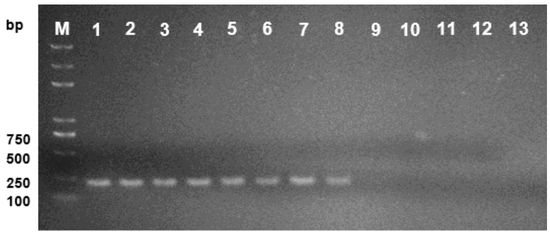

Figure 6. Optimization of the ananealing temperature. Lane 1–12 are results from the annealing temperature of 50, 50.7, 51.7, 53.1, 54.7, 56.4, 58.1, 59.8, 61.4, 62.7, 63.8, and 64.4 °C, respectively in

Commented [M23]: We changed hyphen into endash. Please confirm this revision.

reactions using DNA template of *D. citri* isolate NFHF-8-4. Lane 13 is the ddH2O as the template and lane M, 100 bp ladder.

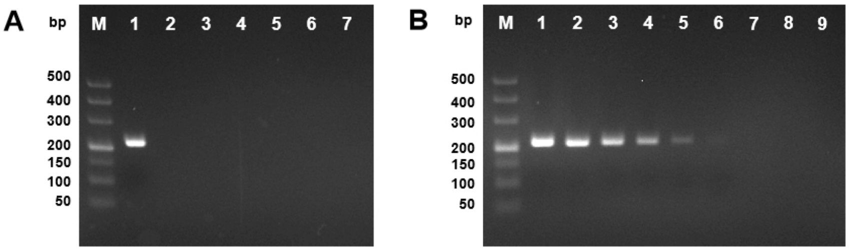

Figure 7. Specificity and sensitivity of the developed PCR based on *TUB* sequence for detection of *D. citri*. (**A**) PCR product 217 bp of *D. citri* (NFHF-8-4) was shown with 2% gel electrophoresis (lane 1). Lanes 2–6 are representatives of *D. citriasiana* (XFAL-1-1), *D. discoidispora* (NKDL-1-2), *D. eres* (NFIF-1-1), *D. sojae* (NFGL-1-5), and *D. unshiuensis* (NFIF-1-6), respectively, Lane 7 is the double sterile water (ddH2O) as negative control, and lane M, 50 bp ladder. (**B**) Sensitivity was investigated with a gDNA serial dilution. Lane 1–8 are gDNA of 10^2, 10^1, 10^0, 10^{-1}, 10^{-2}, 10^{-3}, 10^{-4}, and 0 ng in 20 µL reaction mixture, respectively. Lane 9 is the ddH2O as negative control and lane M, 50 bp ladder.

Commented [M24]: We changed hyphen into endash. Please confirm this revision.

Commented [M25]: We changed hyphen into endash. Please confirm this revision.

3. Discussion

D. citri, a phytopathogenic fungus causing melanose disease has become one of the most devastating citrus pathogens. According to data recorded, the geographic distribution of *D. citri* has been documented in Asia (China, Japan, and Korea), New Zealand, Portugal (Azores Islands), and USA. Even without the DNA sequence database, *D. citri* has also been reported in many other countries, e.g., Brazil, Cambodia, Cuba, Cook Islands, Cote d'Ivoire, Dominican, Haiti, Panama, Puerto Rico, Trinidad and Tobago, Venezuela, Mexico, Fiji, Mauritius, Philippines, Thailand, Myanmar, Niue, Samoa, Tonga, Zimbabwe, and Cyprus. In China, *D. citri* has been documented in several citrus plantations, e.g., Chongqing, Guangxi, Hunan, Jiangxi, Zhejiang, Hong Kong, and Taiwan [21,47–50].

For *Diaporthe* species identification, Santos, et al. [38] suggested the combined multi-locus sequences of ITS, *TEF*, *TUB*, *CAL*, and *HIS*, which were highly effective for resolving boundaries of *Diaporthe* species. Also, a single locus *TEF* gave better delimitation for *Diaporthe* species in phylogeny analysis [38]. Nevertheless, more accurate identification could be obtained based on the combined sequences from *TUB*, *CAL*, *HIS*, and *ITS* loci [38]. It has been reported that several *Diaporthe* species could be confusing, and conflicting results could be observed if only ITS region was used to construct phylogenetic tree [6,39,51]. The *D. citri* strains were isolated from citrus in China and USA, and pathogenicity test confirmed that *D. citri* was the causal agent of melanose and stem-end rot of citrus plant [21,32,33]. However, one cluster named as *D. citri* appeared conflict demonstration with the multi-gene phylogenetic analysis [6,21]. Guarnaccia and Crous [20] analyzed *Diaporthe* species emerging on citrus in European countries and reconsidered that three isolates which were previously recognized as *D. citri*, should be the *D. infertilis* because they were obviously different from other clusters of *D. citri* based on the phylogenetic analysis. In current study, strong evidence with concatenated multi-locus sequences also showed that *D. infertilis* was distinct with *D. citri*. To date, *D. infertilis* has been found on *C. sinensis* (Suriname), *Glycine max* (Brazil), unknown host (Italy), *Citrus limon* (India), and *Mikania glomerate* (Brazil), respectively.

In earlier studies, methods based on PCR were developed for detecting fungal pathogens on citrus. For instance, Bonants, et al. [52] designed species-specific primers from the ITS region to detect *Phyllosticta citricarpa*, a black spot pathogen of orange (*Citrus sinensis*), and lemon (*C. limon*). Wang, et al. [53] also designed species-specific primer pair from ITS to detect black spot disease of pumelo (*C. maxima*). Also, simple PCR was developed to distinguish *Phyllosticta citricarpa* from *Phyllosticta mangiferae* by directly using fungal mycelia on PDA or fruit lesions [54,55]. Real-time PCR

with TaqMan probe was developed for routine quarantine of citrus black spot disease [56]. Similarly, real-time PCR based on ITS was used to distinguish *Phyllosticta citricarpa* from *Phyllosticta citriasiana*, both species could not be distinguished from each other based on morphological characterization [57].

SCAR-marker was developed to detect *Pseudofabraes citricarpa*, a fungus causing target spot on Satsuma mandarin (*Citrus unshiu*) and kumquat (*Fortunella margarita*) in China [58]. Similarly, SACR-marker derived from random amplified polymorphic DNA (RAPD) was used to simultaneously detect *Phytophthora nicotianae* and *Candidatus* Liberibacter asiaticus, the causal agents of citrus roots rot and greening [59]. Pereira, et al. [60] developed a multiplex real-time PCR assay to detect *Colletotrichum abscissum* and *Colletotrichum gloeosporioides*, the causal agents of citrus post-bloom fruit drop.

Latent infected *D. citri* may be the initial source of inoculum of melanose, and a rapid and sensitive diagnosis for detection of this pathogen is currently limited. In previously study, a conserved ITS region was used to design a molecular detection on *D. longicolla*, *D. azadirachtae*, and *D. sclerotioides* [42–45]. Several studies reported that molecular detection of *Diaporthe* species from conserved ITS region was weak and poor, thus could not distinguish the *Diaporthe* complex species [38]. A specific gene *TEF* was used to detect *D. azadirachtae* [46]. However, the molecular tool for *D. citri* detection has not been published. In present study, the novel species-specific PCR assay for detection of *D. citri* was established. This tool can be useful for routine diagnostic work and would be useful to monitor the prevalence of the *D. citri*.

4. Materials and Methods

4.1. Sample Collection and Fungal Isolation

Leaf, fruit, and twig tissues with melanose symptomatic sweet orange (*Citrus sinensis*) and nanfengmiju mandarin (*C. reticulata* cv. *Nanfengmiju*) were collected from Ganzhou city (Xinfeng, Nankang) and Fuzhou city (Nanfeng) Jiangxi Province, China. The samples were collected and took back to Key Lab of Horticultural Plant Biology, Ministry of Education, Huazhong Agricultural University, Wuhan, China. Photos of the diseased samples were captured by using Cannon 600D digital camera (Cannon Inc., Tokyo, Japan). Isolates of *Diaporthe*-like species were isolated from two citrus cultivars, sweet orange and nanfengmiju mandarin showing melanose symptoms in Jiangxi Province, China. Pure isolates were obtained by cutting off the hyphal tips growing from surface-sterilized diseased material. For fungal isolation, each sample of symptomatic tissues was cut into small pieces (5 × 5 mm) with the junction of diseased and healthy tissues. Small pieces of plant tissues were soaked in 75% ethanol solution for 1 min, surface disinfected in 1% sodium hypochlorite solution (NaClO) for 1 min, then rinsed three times with double sterilized water, and dried on sterile tissue paper. Dried small pieces of plant tissues were placed onto potato dextrose agar medium (PDA) amended with 100 µg/mL streptomycin and 100 µg/mL ampicillin (PDA-SA), then incubated for 2–5 days at 25 °C. After that, mycelium tips growing from small pieces of plant tissues were harvested and transferred to Petri dishes with fresh PDA medium for sporulation at 25 °C for 20–30 days. Monosporic isolation was performed according to the method by Goh [61] and Yin, et al. [62]. Pure fungal isolates were kept at 4 °C whenever they are used.

4.2. Geographic Distribution of D. citri

Extensive information of *D. citri* with geographic distribution and host-fungus relationships were investigated in the Systematic Mycology and Microbiology Laboratory, ARS, USDA (SMML database: https://nt.ars-grin.gov/fungaldatabases/https://nt.ars-grin.gov/fungaldata bases/) [63].

4.3. DNA Extraction from Fungal Mycelia

For genomic DNAs (gDNAs) extraction, fresh fungal mycelia were harvested from 7-day old culture on PDA [21]. A hyphal plug about 1.5 square centimeters was cut off and placed into a 2 mL micro-tube with 200 mg of sterile stainless-steel beads (1.6 mm in diameter). Next, 500 µL gDNAs

Commented [M26]: We changed hyphen into endash. Please confirm this revision.

Commented [M27]: We changed hyphen into endash. Please confirm this revision.

Commented [M28]: This URL can not be opened. Please check and edit

extraction lysis buffer (Lysis buffer stock 200 mL: 14.91 g of KCl, 20 mL of 1 M Tris-HCl (pH 8.0), 0.74 g of EDTA-$Na_2 \cdot 2H_2O$ (pH 8.0), adjust with sterile water to 200 mL) was added into the micro-tube. The micro-tube was vigorously homogenized at maximum speed for 10 min on the Bullet Blender® Storm 24 (BBY24M; Next Advance, Inc., New York, NY, USA), then centrifuged at 12,500× *g* for 6 min. Three hundred microliters of gDNAs supernatant were transferred to a new 1.5 mL micro-tube and 300 μL isopropyl alcohol was added. Then, the mixture was gently mixed at room temperature. The solution was centrifuged at 12,500× *g* for 6 min. After discarded the supernatant, gDNAs pellets were rinsed twice with 300 μL of 70% ethanol, and air dried. At last, 30 μL of sterile water (ddH_2O) was added to dissolve gDNAs pellets following Chi's protocol [64]. The gDNAs quality and quantity were measured via UV absorption at wavelength 260 and 280 nm by Thermo Scientific™ NanoDrop 2000 (Thermo Fisher Scientific Inc., Massachusetts, Waltham, MA, USA). The gDNAs was either used or stored at −20 °C until further processing.

4.4. Sequencing of PCR Products

Fragments of nuclear ribosomal internal transcribed spacer regions (ITS), translation elongation factor 1-*α* gene (*TEF*), beta-tubulin gene (*TUB*), calmodulin gene (*CAL*), and histone-3 gene (*HIS*) were amplified by polymerase chain reaction (PCR) with primers described in Table 3. Twenty microliter PCR reaction volume including 1 μL gDNA, 0.8 μL (10 μM) of each primer, 7.6 μL ddH_2O and 10 μL 2 × Hieff® PCR Master Mix (Yeasen Biotech Co., Ltd., Shanghai, China), in a T100™ Thermal Cycler (Bio-Rad, California, Hercules, CA, USA). The PCR reaction was performed following conditions: 95 °C for 3 min, followed by 35 cycles at 95 °C for 30 s, annealing for 50 s at different temperature for different loci, 72 °C for 2 min, and 72 °C for 5 min. The PCR products were applied to electrophoresis in 1% agarose gel and visualized by staining the gel with GoldenView™ dye (Aidlab Biotechnologies Co., Ltd., Beijing, China). The Sanger sequencing of PCR products was performed on ABI 3730xl DNA Sequencer at Wuhan Tianyi Huiyuan Biotechnology Co., Ltd. (Wuhan, China).

Table 3. Universal and species-specific primers used in PCR reactions with *Diaporthe* spp.

Primer Name	Primer Sequences (5′ to 3′)	Length (nt) [1]	Ta (°C) [2]	%GC	Reference
ITS1	TCCGTAGGTGAACCTGCGG	19	55.0	63.2	White, et al. [65]
ITS4	TCCTCCGCTTATTGATATGC	20		45.0	White, et al. [65]
EF1-728F	CATCGAGAAGTTCGAGAAGG	20	58.0	50.0	Carbone and Kohn [66]
EF1-986R	TACTTGAAGGAACCCTTACC	20		45.0	Carbone and Kohn [66]
Bt2a	GGTAACCAAATCGGTGCTGCTTTC	24	58.0	50.0	Glass and Donaldson [67]
Bt2b	ACCCTCAGTGTAGTGACCCTTGGC	24		58.0	Glass and Donaldson [67]
TUBDcitri-F1	CCATTTGACCATCTGCAACAT	21	55.0	42.9	*This study*
TUBD-R1	CCTTGGCCCAGTTGTTTCC	19		57.9	*This study*
CAL-228F	GAGTTCAAGGAGGCCTTCTCCC	22	55.0	59.0	Carbone and Kohn [66]
CAL-737R	CATCTTCTGGCCATCATGG	19		52.6	Carbone and Kohn [66]
CYLH3F	AGGTCCACTGGTGGCAAG	18	58.0	61.1	Crous, et al. [68]
H3-1b	GCGGGCGAGCTGGATGTCCTT	21		66.6	Glass and Donaldson [67]

[1] Number of nucleotides. [2] Annealing temperature estimated by Primer Premier v.6.0.

4.5. Phylogenetic Analyses of Diaporthe Species

Phylogenetic analysis was carried out by using sequences obtained in current study and those downloaded from NCBI's GenBank (www.ncbi.nlm.nih.gov). *Diaporthella corylina* (CBS 121124) was selected as an outgroup (Table 4). All unique DNA sequences were consensus and edited with DNASTAR Lasergene Core Suite software programme (SaeqMan v.7.1.0; DNASTAR Inc., Madison, WI, Wisconsin, USA). Sequences combined different loci were aligned using Clustal W program with supplement software package in BioEdit v.7.2.5 [69]. Maximum parsimony (MP) analysis was done by using PAUP (Phylogenetic Analysis Using Parsimony, v.4.0b10) [70]. The goodness of fit values including tree length (TL), consistency index (CI), retention index (RI), rescaled consistency index (RC), and homoplasy index (HI) were calculated for parsimony and the bootstrap analyses [71]. The heuristic search function was used with 1000 random stepwise addition replicates, with tree bisection and

Commented [M29]: Newly added information, please confirm.

Commented [M30]: Newly added information, please confirm.

Commented [M31]: Newly added information, please confirm.

reconnection (TBR) branch-swapping algorithm, with all characters weighted equally weighted and alignment gaps treated as missing data. Posterior probabilities (PP) were determined using Markov chain Monte Carlo (MCMC) sampling for Bayesian inference (BI) analysis in MrBayes v.3.2.2 [72]. MrModeltest v.2.3 [73] was used to perform statistical selection of the best-fit model of nucleotide substitution with corrected Akaike information criterion (AIC). BI analyses were launched with six simultaneous Markov chains which were run for 105 generations, and trees were sampled every 100th generation (resulting in 10,000 total trees). The calculation of BI analyses was stopped when the average standard deviation of split frequencies fell below 0.01. The consensus trees and posterior probabilities (PP) values were calculated after discarding the first 2000 resulted trees of the analyses as burn-in phase. Finally, above 8000 trees were summarized to calculate the PP in the majority rule consensus tree. Phylogenetic trees were visualized and annotated in FigTree v.1.4.2 [74]. The concatenated alignments and phylogenetic trees were deposited in TreeBASE (study no. S25607), new sequences obtained in this study were submitted to NCBI's GenBank nucleotide database.

Table 4. List of *Diaporthe* species used for phylogenetic analyses.

Species	Isolate Number [1,2]	Plant Host	Locality	GenBank Accession Numbers [3]					Reference(s)
				ITS	*TUB*	*TEF*	*CAL*	*HIS*	
Diaporthe arecae	CBS 161.64 [IT]	*Areca catechu*	Unknown	KC343032	KC344000	KC343758	KC343274	KC343516	Gomes, et al. [6]
	CBS 535.75	*Citrus* sp.	Suriname	KC343033	KC344001	KC343759	KC343275	KC343517	Gomes, et al. [6]
	ZJUD58	*Citrus limon*	China: Yunnan	KJ490593	KJ490414	KJ490472	–	KJ490535	Huang, et al. [48]
	ZJUD59	*Citrus sinensis*	China: Jiangxi	KJ490594	KJ490415	KJ490473	–	KJ490536	Huang, et al. [48]
D. baccae	CBS 136,972 [T]	*Vaccinium corymbosum*	Italy: Sicily, Catania	KJ160565	MF418509	KJ160597	–	MF418264	Guarnaccia and Crous [20], Lombard, et al. [76]
	CPC 26170	*Citrus sinensis*	Italy: Catania	MF418351	MF418510	MF418430	MF418185	MF418265	Guarnaccia and Crous [20]
	CPC 26465	*Citrus limon*	Italy: Catania	MF418352	MF418511	MF418431	MF418186	MF418266	Guarnaccia and Crous [20]
	CPC 26963	*Citrus paradisi*	Italy: Vibo Valentia	MF418353	MF418512	MF418432	MF418187	MF418267	Guarnaccia and Crous [20]
	CPC 27821	*Citrus reticulata*	Italy: Cosenza	MF418357	MF418516	MF418436	MF418191	MF418271	Guarnaccia and Crous [20]
D. biconispora	CGMCC3.17252 [T]	*Citrus grandis*	China: Fujian	KJ490597	KJ490418	KJ490476	–	KJ490539	Huang, et al. [48]
	ZJUD60	*Citrus sinensis*	China: Jiangxi	KJ490595	KJ490416	KJ490474	–	KJ490537	Huang, et al. [48]
	ZJUD61	*Fortunella margarita*	China: Guangxi	KJ490596	KJ490417	KJ490475	–	KJ490538	Huang, et al. [48]
D. biguttulata	CGMCC3.17248 [T]	*Citus limon*	China: Yunnan	KJ490582	KJ490403	KJ490461	–	KJ490524	Huang, et al. [48]
	ZJUD48	*Citrus limon*	China: Yunnan	KJ490583	KJ490404	KJ490462	–	KJ490525	Huang, et al. [48]
D. citri	AR3405 [T]	*Citrus* sp.	USA: Florida	KC843311	KC843187	KC843071	KC843157	MF418281	Guarnaccia and Crous [20], Udayanga, et al. [24]
	CBS 134,239 [T]	*Citrus sinensis*	USA: Florida	KC357553	KC357456	KC357522	KC357488	MF418280	Guarnaccia and Crous [20], Huang, et al. [21]
	ZJUD1	*Citrus reticulata*	China: Zhejiang	JQ954654	KJ490395	JQ954671	–	KJ490514	Huang, et al. [21], Huang, et al. [48]
	CBS 144227	*Citrus reticulata*	Portugal: Azores	MH063904	MH063916	MH063910	MH063892	MH063898	Guarnaccia and Crous [20]
	CBS 135426	*Citrus unshiu* cv. *Juwadeun*	Korea: Odeung-dong	KC843324	KC843200	KC843084	KC843170	–	Udayanga, et al. [24]
	ICMP 10355	*Citrus reticulata*	New Zealand: Kerikeri	KC843314	KC843190	KC843074	KC843160	–	Udayanga, et al. [24]
	Ph-18	*Citrus sinensis*	Panama: Coclé	MK214464	–	MK283703	–	–	Aguilera-Cogley and Vicent [77]
	FCDC2	*Citrus* sp.	Japan: Fukuoka	AB302249	–	–	–	–	Kanematsu, et al. [78], Kanematsu [79]
D. citriasiana	CGMCC3.15224 [T]	*Citrus unshiu*	China: Shaanxi	JQ954645	KC357459	JQ954663	KC357491	MF418282	Guarnaccia and Crous [20], Huang, et al. [21]
	ZJUD33	*Citrus paradisi*	China: Jiangxi	JQ954658	KC357460	JQ972716	KC357493	–	Huang, et al. [21]
	ZJUD81	*Citrus grandis* cv. *Shatianyou*	China: Zhejiang	KJ490616	KJ490437	KJ490495	–	KJ490558	Huang, et al. [48]
D. citrichinensis	CGMCC3.15225 [T]	*Citrus unshiu*	China: Shaanxi	JQ954648	MF418524	JQ954666	KC357494	KJ490516	Guarnaccia and Crous [20], Huang, et al. [21,48]
	ZJUD034B	*Citrus unshiu*	China: Shaanxi	KJ210539	KJ420829	KJ210562	KJ435042	KJ420879	Udayanga, et al. [24], Udayanga, et al. [39]
	ZJUD38	*Citrus unshiu*	China: Shaanxi	KC357558	KC357463	KC357527	KC357498	–	Huang, et al. [21]
	ZJUD85	*Fortunella margarita*	China: Guangxi	KJ490620	KJ490441	KJ490499	–	KJ490562	Huang, et al. [48]
	ZJUD96	*Citrus unshiu*	China: Fujian	KJ490631	KJ490452	KJ490510	–	KJ490573	Huang, et al. [48]
	ZJUD97	*Citrus grandis*	China: Fujian	KJ490632	KJ490453	KJ490511	–	KJ490574	Huang, et al. [48]
D. cytosporella	CBS 137,020 [T]	*Citrus limon*	Spain	KC843307	KC843221	KC843116	KC843141	MF418283	Guarnaccia and Crous [20], Udayanga, et al.

									[24]
	AR5149	*Citrus sinensis*	USA: California	KC843309	KC843222	KC843118	KC843143	–	Udayanga, et al. [24]
D. discoidispora	CGMCC3.17255 [T]	*Citrus unshiu*	China: Jiangxi	KJ490624	KJ490445	KJ490503	–	KJ490566	Huang, et al. [48]
D. endophytica	CBS 133,811 [T]	*Schinus terebinthifolius*	Brazil	KC343065	KC344033	KC343791	KC343307	KC343549	Gomes, et al. [6]
	ZJUD73	*Citrus unshiu*	China: Fujian	KJ490608	KJ490429	KJ490487	–	KJ490550	Huang, et al. [48]
	ZJUD94	*Citrus limon*	China: Yunnan	KJ490629	KJ490450	KJ490508	–	KJ490571	Huang, et al. [48]
D. eres	CGMCC3.17081 [T]	*Lithocarpus glabra*	China: Zhejiang	KF576282	KF576306	KF576257	–	–	Gao, et al. [80]
	CGMCC3.17089 [T]	*Lithocarpus glabra*	China: Zhejiang	KF576267	KF576291	KF576242	–	–	Gao, et al. [80]
	ZJUD84	*Fortunella margarita*	China: Guangxi	KJ490619	KJ490440	KJ490498	–	KJ490561	Huang, et al. [48]
	ZJUD90	*Citrus unshiu*	China: Jiangxi	KJ490625	KJ490446	KJ490504	–	KJ490567	Huang, et al. [48]
	ZJUD91	*Citrus* sp.	China: Jiangxi	KJ490626	KJ490447	KJ490505	–	KJ490568	Huang, et al. [48]
	ZJUD92	*Citrus* sp.	China: Zhejiang	KJ490627	KJ490448	KJ490506	–	KJ490569	Huang, et al. [48]
D. foeniculina	CBS 123,208 [T]	*Foeniculum vulgare*	Portugal: Évora	KC343104	KC344072	KC343830	KC343346	KC343588	Gomes, et al. [6]
	CBS 135430	*Citrus limon*	USA: California	KC843301	KC843215	KC843110	KC843135	MF418284	Guarnaccia and Crous [20], Udayanga, et al. [24]
	CPC 26184	*Citrus maxima*	Italy: Messina	MF418365	MF418525	MF418444	MF418199	MF418285	Guarnaccia and Crous [20]
	CPC 26885	*Citrus bergamia*	Greece: Missolonghi	MF418374	MF418534	MF418453	MF418208	MF418294	Guarnaccia and Crous [20]
	CPC 26967	*Citrus mitis*	Italy: Messina	MF418379	MF418539	MF418458	MF418213	MF418299	Guarnaccia and Crous [20]
	CPC 27895	*Citrus japonica*	Malta: Gozo	MF418391	MF418551	MF418470	MF418225	MF418311	Guarnaccia and Crous [20]
	CPC 27945	*Citrus paradisi*	Portugal: Faro	MF418397	MF418557	MF418476	MF418231	MF418317	Guarnaccia and Crous [20]
	CPC 28033	*Citrus sinensis*	Portugal: Mesquita	MF418402	MF418562	MF418481	MF418236	MF418322	Guarnaccia and Crous [20]
	CPC 28081	*Citrus reticulata*	Spain: Algemesi	MF418415	MF418575	MF418494	MF418249	MF418335	Guarnaccia and Crous [20]
	CPC 28163	*Microcitrus australasica*	Italy: Catania	MF418416	MF418576	MF418495	MF418250	MF418336	Guarnaccia and Crous [20]
D. hongkongensis	HKUCC 9104 [T]	*Dichroa febrifuga*	Hong Kong: China	KC343119	KC344087	KC343845	KC343361	KC343603	Gomes, et al. [6]
	ZJUD74	*Citrus unshiu*	China: Fujian	KJ490609	KJ490430	KJ490488	–	KJ490551	Huang, et al. [48]
	ZJUD75	*Citrus reticulata*	China: Fujian	KJ490610	KJ490431	KJ490489	–	KJ490552	Huang, et al. [48]
	ZJUD76	*Citrus reticulata* cv. *Nanfengmiju*	China: Jiangxi	KJ490611	KJ490432	KJ490490	–	KJ490553	Huang, et al. [48]
	ZJUD77	*Citrus unshiu*	China: Zhejiang	KJ490612	KJ490433	KJ490491	–	KJ490554	Huang, et al. [48]
	ZJUD78	*Citrus grandis*	China: Fujian	KJ490613	KJ490434	KJ490492	–	KJ490555	Huang, et al. [48]
	ZJUD79	*Citrus grandis*	China: Fujian	KJ490614	KJ490435	KJ490493	–	KJ490556	Huang, et al. [48]
D. infertilis	CBS 230.52 [T]	*Citrus sinensis*	Suriname: Paramaribo	KC343052	KC344020	KC343778	KC343294	KC343536	Gomes, et al. [6]
	CBS 199.39	Unknown	Italy	KC343051	KC344019	KC343777	KC343293	KC343535	Gomes, et al. [6]
	CPC 20322	*Glycine max*	Brazil	KC343053	KC344021	KC343779	KC343295	KC343537	Gomes, et al. [6]
	Pc4	*Citrus limon*	India	KJ477016	–	–	–	–	Mahadevakumar, et al. [81]
	G-01	*Mikania glomerata*	Brazil	KJ934221	KT962837	KT962838	–	–	Polonio, et al. [82], Polonio, et al. [83]
	G-02	*Mikania glomerata*	Brazil	KJ934219	–	–	–	–	Polonio, et al. [82]
	G-03	*Mikania glomerata*	Brazil	KJ934220	–	–	–	–	Polonio, et al. [82]
D. limonicola	CBS 142,549 [T]	*Citrus limon*	Malta: Gozo	MF418422	MF418582	MF418501	MF418256	MF418342	Guarnaccia and Crous [20]
	CPC 31137	*Citrus limon*	Malta: Zurrieq	MF418423	MF418583	MF418502	MF418257	MF418343	Guarnaccia and Crous [20]
D. melitensis	CBS 142,551 [T]	*Citrus limon*	Malta: Gozo	MF418424	MF418584	MF418503	MF418258	MF418344	Guarnaccia and Crous [20]

	CPC 27875	*Citrus limon*	Malta: Gozo	MF418425	MF418585	MF418504	MF418259	MF418345	Guarnaccia and Crous [20]
D. multigutullata	CGMCC3.17258 [T]	*Citrus grandis*	China: Fujian	KJ490633	KJ490454	KJ490512	–	KJ490575	Huang, et al. [48]
D. novem	CBS 127,270 [T]	*Glycine max*	Croatia	KC343156	KC344124	KC343882	KC343398	KC343640	Gomes, et al. [6]
	CPC 26188	*Citrus japonica*	Italy: Messina	MF418426	MF418586	MF418505	MF418260	MF418346	Guarnaccia and Crous [20]
	CPC 28165	*Citrus aurantiifolia*	Italy: Catania	MF418427	MF418587	MF418506	MF418261	MF418347	Guarnaccia and Crous [20]
	CPC 28167	*Citrus aurantiifolia*	Italy: Catania	MF418428	MF418588	MF418507	MF418262	MF418348	Guarnaccia and Crous [20]
	CPC 28169	*Citrus aurantiifolia*	Italy: Catania	MF418429	MF418589	MF418508	MF418263	MF418349	Guarnaccia and Crous [20]
D. ovalispora	CGMCC3.17256 [T]	*Citrus limon*	China: Yunnan	KJ490628	KJ490449	KJ490507	–	KJ490570	Huang, et al. [48]
D. sojae	CBS 139,282 [ET]	*Glycine max*	USA: Ohio	KJ590719	KJ610875	KJ590762	KJ612116	KJ659208	Udayanga, et al. [51]
	ZJUD68	*Citrus unshiu*	China: Zhejiang	KJ490603	KJ490424	KJ490482	–	KJ490545	Huang, et al. [48]
	ZJUD69	*Citrus reticulata* cv. *Nanfengmiju*	China: Jiangxi	KJ490604	KJ490425	KJ490483	–	KJ490546	Huang, et al. [48]
	ZJUD70	*Citrus limon*	China: Yunnan	KJ490605	KJ490426	KJ490484	–	KJ490547	Huang, et al. [48]
	ZJUD71	*Citrus reticulata*	China: Zhejiang	KJ490606	KJ490427	KJ490485	–	KJ490548	Huang, et al. [48]
	ZJUD72	*Citrus reticulata*	China: Yunnan	KJ490607	KJ490428	KJ490486	–	KJ490549	Huang, et al. [48]
D. subclavata	CGMCC3.17257 [T]	*Citrus unshiu*	China: Fujian	KJ490630	KJ490451	KJ490509	–	KJ490572	Huang, et al. [48]
	ZJUD83	*Citrus grandis* cv. *Shatianyou*	China: Guangdong	KJ490618	KJ490439	KJ490497	–	KJ490560	Huang, et al. [48]
D. unshiuensis	CGMCC3.17569 [T]	*Citrus unshiu*	China: Zhejiang	KJ490587	KJ490408	KJ490466	–	KJ490529	Huang, et al. [48]
	CGMCC3.17566	*Fortunella margarita*	China: Guilin	KJ490584	KJ490405	KJ490463	–	KJ490526	Huang, et al. [48]
	CGMCC3.17567	*Fortunella margarita*	China: Guilin	KJ490585	KJ490406	KJ490464	–	KJ490527	Huang, et al. [48]
	CGMCC3.17568	*Fortunella margarita*	China: Guilin	KJ490586	KJ490407	KJ490465	–	KJ490528	Huang, et al. [48]
Diaporthella corylina	CBS 121,124 [T]	*Corylus* sp.	China: Heilongjiang	KC343004	KC343972	KC343730	KC343246	KC343488	Gomes, et al. [6], Vasilyeva, et al. [84]

[1] IT = ex-isotype, T = ex-type, and EP = ex-epitype. [2] AR = Corresponding author's personal collection of A.Y. Rossman; CBS = Westerdijk Fungal Biodiversity Institute (formerly CBSKNAW), Utrecht, The Netherlands; CFCC = China Forestry Culture Collection Center, China; CGMCC = China General Microbiological Culture Collection, China; CPC = Culture collection of P.W. Crous, housed at Westerdijk Fungal Biodiversity Institute, Utrecht, The Netherlands; HKUCC = University of Hong Kong Culture Collection, Department of Ecology and Biodiversity, Hong Kong, China; ICMP = International Collection of Micro-organisms from Plants, Auckland, New Zealand; and ZJUD = *Diaporthe* species culture collection at the Institute of Biotechnology, Zhejiang University, Hangzhou, China. [3] ITS = nuclear ribosomal internal transcribed spacer regions; *TUB* = beta-tubulin gene; *TEF* = translation elongation factor 1-α gene; *HIS* = histone-3 gene; and *CAL* = calmodulin gene.

4.6. Morphology and Culture Characteristics of *D. citri*

Isolates on PDA plates were incubated at 25 °C for 30 days under near-ultraviolet (UV) light (12 h light/12 h dark). The growth rate of mycelium was measured in five duplicates. Colony color on PDA, Corn meal agar (CMA), and Oatmeal agar (OMA) media incubated at 25 °C near UV light with 12 h, was investigated according to the method of Rayner [74~~5~~]. The morphology imagines were taken using Canon 600D digital camera (Canon Inc., Tokyo, Japan) after 10 days of incubation. Conidiomata and conidia were observed under the OLYMPUS SZX16 stereomicroscope (Olympus Corporation, Tokyo, Japan), conidial length/wide ratio of 30 conidia was measured with a stage micrometer under a Motic BA200 light microscope (Motic China Group Co., Ltd., Nanjing, China). Alpha and beta conidia were measured for calculating means ($\bar{x}$) and standard deviations (SD). The conidia ranges were shown as (min–) $\bar{x}$ − SD − $\bar{x}$ + SD (−max) μm ($\bar{x}$ ± SD). Conidia digital images were captured using Nikon Eclipse 80i compound light microscope imaging system (Nikon Corporation, Tokyo, Japan).

4.7. Primer Design and Development of the Molecular Tool to Detect *D. citri*

A highly varied region in *TUB* gene was selected as the target for developing molecular tool based on PCR to specifically detect *D. citri* from other *Diaporthe* species. Partial *TUB* gene of *D. citri* was retrieved from NCBI GenBank database (accession no. MN894459). The obtained sequences were aligned by using Clustal W algorithm in software package BioEdit v.7.2.5 [69]. The primers were designed by analyzing hairpin-dimer potential, length of the desired amplicon, %GC content, and melting temperatures (Ta) in Primer premier 6.0 software (Premier Biosoft International, ~~Palo Alto,~~California, ~~CA,~~ USA). The primers were synthesized by Wuhan Tianyi Huiyuan Biotechnology Co., Ltd. (Wuhan, China). All the primer sequences used in this study are listed in Table 3.

Firstly, the annealing temperature was optimized in a gradient PCR in which the annealing temperatures were set from 50 to 65 °C. For specificity evaluation, gDNAs of *D. citri* (NFHF-8-4), *D. citriasiana* (XFAL-1-1), *D. discoidispora* (NKDL-1-2), *D. eres* (NFIF-1-1), *D. sojae* (NFGL-1-5), and *D. unshiuensis* (NFIF-1-6) were used, because these species are the closely related *Diaporthe* species in the phylogenetic analysis. The PCR reaction was performed in a final volume of 20 μL with the following components: 10 μL 2 × Hieff® PCR Master Mix (Yeasen Biotech Co., Ltd., Shanghai, China), 7.6 μL ddH_2O, 0.8 μL (10 μM) of each species-specific primer (TUBDcitri-F1/TUBD-R1), and 1 μL gDNA (10 ng). The T100™ Thermal Cycler (Bio-Rad, USA) was programmed for conditions as 95 °C for 3 min, followed by 35 cycles at 95 °C for 30 s, annealing temperature (Ta) of 55 °C for 2 min, and 72 °C for 5 min. Finally, 5 μL products were used to electrophoresis on 2% agarose gel and visualized by staining the gel with GoldenView™ dye (Aidlab Biotechnologies Co., Ltd., Beijing, China), along with a 50 bp ladder as molecular marker (*GL* DNA Marker 500; Accurate Biotechnology (Hunan) Co., Ltd., Hunan, China) and 100 bp ladder (DNA 2K plus marker; TransGen Biotech Co., Ltd., Beijing, China). Similar test was also applied for the phylogenetically analyzed 38 isolates. For sensitivity evaluation, a serial of 10-fold dilutions of gDNA from *D. citri* isolate NFHF-8-4 ranging from 10^2 to 10^{-4} ng in 20 μL reaction mixture were used under the conditions described above.

5. Conclusions

In current study, it has been documented that *Diaporthe* species could cause devastating citrus diseases and *D. citri* was the causal agent of the citrus melanose disease. Based on the phylogenetic analysis with five multi-locus sequences, *Diaporthe* species boundaries could be clearly delimitated. We also designed species-specific primers from *TUB* gene to develop PCR method for detecting *D. citri*. The PCR-based method showed high specificity and sensitivity, that could be applied for detection of *D. citri* efficiently in practice. In the future, efficient PCR should be developed with citrus tissues infected by *D. citri* and multiple PCR which can distinguish different *Diaporthe* species should be developed for the phytosanitary assay in plant quarantine routine work.

Commented [M32]: Reference order is wrong. Ref 75 should be cited after 74.

Commented [ซไ33]: Confirmed Rayner [74]

Commented [M34]: Newly added information, please confirm.

Commented [ซไ35]: confirmed

Commented [M36]: We changed hyphen into minus. Please confirm this revision.

Supplementary Materials: The following are available online at www.mdpi.com/xxx/s1, Table S1: Checklist of *Diaporthe citri* and *D. infertilis* associated with details citrus host and their allied genera, locality and their reference(s), Figure S1: Phylogenetic trees of *Diaporthe* spp. by Bayesian inference (BI) analysis based on combined data set and individual locus (ITS, *TUB, TEF, CAL*, and *HIS*, respectively). Ex-type, ex-isotype, and ex-epitype strains are indicated in bold. The species *Diaporthella corylina* (CBS 121124) was selected as an outgroup, Figure S2: Phylogenetic tree of *Diaporthe* spp. generated by Maximum Parsimony (MP) analysis based on combined data set and individual locus (ITS, *TUB*, *TEF*, *CAL*, and *HIS*, respectively). Ex-type, ex-isotype, and ex-epitype strains are indicated in bold. The species *Diaporthella corylina* (CBS 121124) was selected as an outgroup, Figure S3: The prevalence of *Diaporthe* species on citrus in Jiangxi Province, China based on phylogenetic identification. Number (%) indicate the number of obtained isolates of certain species and the percentage among the total 140 isolates. Figure S4: Species-specific 217 bp *TUB* gene amplified by the primer pair TUBDcitri-F1/TUBD-R1 was shown with 2% gel electrophoresis. Thirty-eight representatives that were identified based on phylogenetic analysis were used to confirm the specificity of PCR approach. The numbers of *D. citri, D. citriasiana, D. discoidispora, D. eres, D. sojae,* and *D. unshiuensis* isolates were 10, 3, 5, 10, 5, and 5, respectively. Lane CK is the double sterile water (ddH$_2$O) as negative control and lane M, 100 bp ladder.

Formatted: Subscript

Author Contributions: Conceptualization, C.C., Y.L. and C.-X.L.; Validation, C.C., X.-Y.L., Y.L. and C.-X.L.; Formal analysis, C.C. and X.-Y.L.; Investigation, C.C., X.-Y.L., J.-B.L. and B.X.; Resources, J.-B.L. and B.X.; Data curation, C.C., X.-Y.L., Y.L. and C.-X.L.; Writing, C.C. and C.-X.L.; Funding acquisition, Y.L. and C.-X.L.

Funding: This work was supported by the National Key Research and Development Program of China (No. 2017YFD020200103).

Acknowledgments: We gratefully thank Mingkhuan Doilom (Key Laboratory for Plant Diversity and Biogeography of East Asia, Kunming Institute of Botany, Chinese Academy of Sciences, China) and Sinang Hongsanan (Shenzhen Key Laboratory of Microbial Genetic Engineering, Shenzhen University, China) for technical assistance and valuable advice.

Conflicts of Interest: The authors declare no conflicts of interest. The funders had no role in the design of the study; in the collection, analyses, or interpretation of data; in the writing of the manuscript; or in the decision to publish the results

References

1. Wu, G.A.; Terol, J.; Ibanez, V.; López-García, A.; Pérez-Román, E.; Borredá, C.; Domingo, C.; Tadeo, F.R.; Carbonell-Caballero, J.; Alonso, R.; et al. Genomics of the origin and evolution of *Citrus*. *Nature* **2018**, *554*, 311–330.
2. FAO. *Citrus fruit—Fresh and processed statistical bulletin 2016*; Food and Agriculture Organization of the United Nations: Rome, Italy, 2017.
3. Deng, X.X.; Peng, C.J.; Chen, Z.S.; Deng, Z.N.; Xu, J.G.; Li, J. *Citrus Varieties in China*; China Agriculture Press: Beijing, China, 2008.
4. Boddy, L.; Griffth, G.S. Role of endophytes and latent invasion in the development of decay communities in sapwood of angiospermous trees. *Sydowia* **1989**, *41*, 41–73.
5. Carroll, G.C. *The Biology of Endophytism in Plants with Particular Reference to Woody Perennials*; Cambridge University Press: Cambridge, UK, 1986.
6. Gomes, R.R.; Glienke, C.; Videira, S.I.R.; Lombard, L.; Groenewald, J.Z.; Crous, P.W. *Diaporthe*: A genus of endophytic, saprobic and plant pathogenic fungi. *Persoonia* **2013**, *31*, 1–41.
7. Marin-Felix, Y.; Hernández-Restrepo, M.; Wingfield, M.J.; Akulov, A.; Carnegie, A.J.; Cheewangkoon, R.; Gramaje, D.; Groenewald, J.Z.; Guarnaccia, V.; Halleen, F.; et al. Genera of phytopathogenic fungi: GOPHY 2. *Stud. Mycol.* **2019**, *92*, 47–133.
8. Suryanarayanan, T.S.; Devarajan, P.T.; Girivasan, K.P.; Govindarajulu, M.B.; Kumaresan, V.; Murali, T.S.; Rajamani, T.; Thirunavukkarasu, N.; Venkatesan, G. The host range of multi-host endophytic fungi. *Curr. Sci.* **2018**, *115*, 1963–1969.
9. Guarnaccia, V.; Vitale, A.; Cirvilleri, G.; Aiello, D.; Susca, A.; Epifani, F.; Perrone, G.; Polizzi, G. Characterisation and pathogenicity of fungal species associated with branch cankers and stem-end rot of avocado in Italy. *Eur. J. Plant Pathol.* **2016**, *146*, 963–976.
10. Mostert, L.; Crous, P.W.; Kang, J.C.; Phillips, A.J.L. Species of *Phomopsis* and a *Libertella* sp. occurring on grapevines with specific reference to South Africa: Morphological, cultural, molecular and pathological characterization. *Mycologia* **2001**, *93*, 146–167.

Commented [M37]: Ref 22: Newly added information, please confirm.
Ref 27: Please confirm the journal name and provide correct abbreviation.
Ref 47: Please add the city.
Ref 49: Please revise the author name.
Ref 57, 58: Newly added information and revise, please confirm.
Ref 63, 73, 74: Please add the location of the publisher.
Ref 75: Revise, please confirm.

Commented [ชไ38]: Another Ref was confirmed
For Ref of 47: only it's fine
For Ref of 49: Yanna, have only family name

11. Rehner, S.A.; Uecker, F.A. Nuclear ribosomal internal transcribed spacer phylogeny and host diversity in the coelomycete *Phomopsis*. *Can. J. Bot.* **1994**, *72*, 1666–1674.
12. Santos, J.M.; Vrandečić, K.; Ćosić, J.; Duvnjak, T.; Phillips, A.J.L. Resolving the *Diaporthe* species occurring on soybean in Croatia. *Persoonia* **2011**, *27*, 9–19.
13. Thompson, S.M.; Tan, Y.P.; Young, A.J.; Neate, S.M.; Aitken, E.A.B.; Shivas, R.G. Stem cankers on sunflower (*Helianthus annuus*) in Australia reveal a complex of pathogenic *Diaporthe* (*Phomopsis*) species. *Persoonia* **2011**, *27*, 80–89.
14. Cai, L.; Giraud, T.; Zhang, N.; Begerow, D.; Cai, G.H.; Shivas, R.G. The evolution of species concepts and species recognition criteria in plant pathogenic fungi. *Fungal Divers.* **2011**, *50*, 121–133.
15. Duan, W.J.; Yan, J.; Liu, F.; Cai, L.; Zhu, S.F. The list of Chinese quarantine fungi is in need of revision and renewal (in Chinese). *Mycosystema* **2015**, *34*, 942–960.
16. Rossman, A.Y.; Palm-Hernández, M.E. Systematics of plant pathogenic fungi: Why it matters. *Plant Dis.* **2008**, *92*, 1376–1386.
17. Shivas, R.G.; Cai, L. Cryptic fungal species unmasked. *Microbiol. Aust.* **2012**, *33*, 36–37.
18. Timmer, L.W.; Garnsey, S.M.; Graham, J.H. *Scab Diseases*, revised edition: 31–32 ed.; American Phytopathological Society Press: St. Paul, MN, USA, 2000; p. 92.
19. Whiteside, J.O.; Timmer, L.W. *Citrus Diseases: General Concepts*, revised edition: 3–4 ed.; American Phytopathological Society: St. Paul, MN, USA, 2000.
20. Guarnaccia, V.; Crous, P.W. Emerging citrus diseases in Europe caused by species of *Diaporthe*. *IMA Fungus* **2017**, *8*, 317–334.
21. Huang, F.; Hou, X.; Dewdney, M.M.; Fu, Y.S.; Chen, G.Q.; Hyde, K.D.; Li, H.Y. *Diaporthe* species occurring on citrus in China. *Fungal Divers.* **2013**, *61*, 237–250.
22. Kucharek, T.; Whiteside, J.; Brown, E. *Melanose and Stem End Rot of Citrus*; Florida Cooperative Extension Service, Institute of Food and Agricultural Sciences, University of Florida: Florida~~Gainesville, FL~~, USA, 1983.
23. Mondal, S.N.; Vicent, A.; Reis, R.F.; Timmer, L.W. Saprophytic colonization of citrus twigs by *Diaporthe citri* and factors affecting pycnidial production and conidial survival. *Plant Dis.* **2007**, *91*, 387–392
24. Udayanga, D.; Castlebury, L.A.; Rossman, A.Y.; Hyde, K.D. Species limits in *Diaporthe*: Molecular re-assessment of *D. citri*, *D. cytosporella*, *D. foeniculina* and *D. rudis*. *Persoonia* **2014**, *32*, 83–101.
25. Swingle, W.T.; Webber, H.J. The principal disease of citrus fruits in Florida. *USDA Div. Veg. Physiol. Pathol. Bull.* **1896**, *8*, 9–14.
26. Fawcett, H.S. The cause of stem-end rot of citrus fruits (*Phomopsis citri* n. sp.). *Phytopathology* **1912**, *2*, 109–113.
27. Floyd, B.F.; Stevens, H.E. Melanose and stem-end rot. *Fla. Agr. Expt. Sta. Bull.* **1912**, *111*, 1–16.
28. Rehm, H. Ascomycetes philippinenses VI. *Leafl. Philipp. Bot.* **1914**, *6*, 2258–2281.
29. Horne, W.T. A *Phomopsis* in grape fruit from the isle of Pines W. I., with notes on *Diplodia natalensis*. *Phytopathology* **1922**, *12*, 414–418.
30. Nitschke, T.R.J. Pyrenomycetes germanici. In *Die kernpilze Deutschlands Bearbeitet Von Dr. Th. Nitschke*; Eduard Trewendt: Breslau, Germany, 1870; Volume 2, pp. 161–320.
31. Fawcett, H.S. A *Phomopsis* of citrus in California. *Phytopathology* **1922**, *12*, 107.
32. Bach, W.J.; Wolf, F.A. The isolation of the fungus that causes citrus melanose and the pathological anatomy of the host. *J. Agric. Res.* **1928**, *37*, 243–252.
33. Ruehle, G.D.; Kuntz, W.A. *Melanose of Citrus and Its Commercial Control*; Florida Agricultural Experiment Station Bulletin, University of Florida: Gainesville, FL, USA, 1940.
34. Castlebury, L. The *Diaporthe vaccinii* complex of fruit pathogens. *Inoculum* **2005**, *56*, 12.
35. Santos, J.M.; Correia, V.G.; Phillips, A.J.L.; Spatafora, J.W. Primers for mating-type diagnosis in *Diaporthe* and *Phomopsis*: Their use in teleomorph induction in vitro and biological species definition. *Fungal Biol.* **2010**, *114*, 255–270.
36. Santos, J.M.; Phillips, A.J.L. Resolving the complex of *Diaporthe* (*Phomopsis*) species occurring on *Foeniculum vulgare* in Portugal. *Fungal Divers.* **2009**, *34*, 111–125.
37. Guarnaccia, V.; Groenewald, J.Z.; Woodhall, J.; Armengol, J.; Cinelli, T.; Eichmeier, A.; Ezra, D.; Fontaine, F.; Gramaje, D.; Gutierrez-Aguirregabiria, A.; et al. *Diaporthe* diversity and pathogenicity revealed from a broad survey of grapevine diseases in Europe. *Persoonia* **2018**, *40*, 135–153.

38. Santos, L.; Alves, A.; Alves, R. Evaluating multi-locus phylogenies for species boundaries determination in the genus *Diaporthe*. *PeerJ* **2017**, *5*, 1–26.
39. Udayanga, D.; Castlebury, L.A.; Rossman, A.Y.; Chukeatirote, E.; Hyde, K.D. Insights into the genus *diaporthe*: Phylogenetic species delimitation in the *D. eres* species complex. *Fungal Divers.* **2014**, *67*, 203–229.
40. Yang, Q.; Fan, X.L.; Guarnaccia, V.; Tian, C.M. High diversity of *Diaporthe* species associated with dieback diseases in China, with twelve new species described. *MycoKeys* **2018**, *39*, 97–149.
41. Hyde, K.D.; Nilsson, R.H.; Alias, S.A.; Ariyawansa, H.A.; Blair, J.E.; Cai, L.; de Cock, A.W.A.M.; Dissanayake, A.J.; Glockling, S.L.; Goonasekara, I.D.; et al. One stop shop: Backbones trees for important phytopathogenic genera: I (2014). *Fungal Divers.* **2014**, *67*, 21–125.
42. Zhang, A.W.; Hartman, G.L.; Riccioni, L.; Chen, W.D.; Ma, R.Z.; Pedersen, W.L. Using PCR to distinguish *Diaporthe phaseolorum* and *Phomopsis longicolla* from other soybean fungal pathogens and to detect them in soybean tissues. *Plant Dis.* **1997**, *81*, 1143–1149.
43. Prasad, M.N.N.; Bhat, S.S.; Raj, A.P.C.; Janardhana, G.R. Molecular detection of *Phomopsis azadirachtae*, the causative agent of dieback disease of neem by polymerase chain reaction. *Curr. Sci.* **2006**, *91*, 158–159.
44. Vedashree, S.; Sateesh, M.K.; Chowdappa, P.; Nirmalkumar, B.J. Species-specific PCR-based assay for identification and detection of *Phomopsis* (*Diaporthe*) *azadirachtae* causing die-back disease in *Azadirachta indica*. *J. Phytopathol.* **2015**, *163*, 818–828.
45. Shishido, M.; Sato, K.; Yoshida, N.; Tsukui, R.; Usami, T. PCR-based assays to detect and quantify *Phomopsis sclerotioides* in plants and soil. *J. Gen. Plant Pathol.* **2010**, *76*, 21–30.
46. Shirahatti, P.; Ramu, R.; Purushothama, C.R.A.; Prasad, M.N.N. Development of a simple and reliable species-species detection of *Phomopsis azadirachtae*, using the translation elongation factor 1-alpha gene. *Eur. J. Plant Pathol.* **2015**, *141*, 769–778.
47. Anonymous. *List of Plant Diseases in Taiwan*; Plant Protection Soc: Taichung, China, 1979; p. 404.
48. Huang, F.; Udayanga, D.; Wang, X.H.; Hou, X.; Mei, X.F.; Fu, Y.S.; Hyde, K.D.; Li, H.Y. Endophytic *Diaporthe* associated with *citrus*: A phylogenetic reassessment with seven new species from China. *Fungal Biol.* **2015**, *119*, 331–347.
49. Lu, B.S.; Hyde, K.D.; Ho, W.H.; Tsui, K.M.; Taylor, J.E.; Wong, K.M.; Yanna, Z.D.; Zhou, D.Q. *Checklist of Hong Kong Fungi*; Fungal Diversity Press: Hong Kong, China, 2000; p. 207.
50. Zhuang, W.Y. *Higher Fungi of Tropical China*; Mycotaxon, Ltd.: Ithaca, NY, USA, 2001; p. 485.
51. Udayanga, D.; Castlebury, L.A.; Rossman, A.Y.; Chukeatirote, E.; Hyde, K.D. The *Diaporthe sojae* species complex: Phylogenetic re-assessment of pathogens associated with soybean, cucurbits and other field crops. *Fungal Biol.* **2015**, *119*, 383–407.
52. Bonants, P.J.M.; Carroll, G.C.; de Weerdt, M.; van Brouwershaven, I.R.; Baayen, R.P. Development and validation of a PCR-based detection method for pathogenic isolates of the citrus black spot fungus, *Guignardia citricarpa*. *Eur. J. Plant Pathol.* **2003**, *109*, 503–513.
53. Wang, X.H.; Chen, G.Q.; Huang, F.; Zhang, J.Z.; Hyde, K.D.; Li, H.Y. *Phyllosticta* species associated with citrus diseases in China. *Fungal Divers.* **2012**, *52*, 209–224.
54. Meyer, L.; Sanders, G.M.; Jacobs, R.; Korsten, L. A one-day sensitive method to detect and distinguish between the citrus black spot pathogen *Guignardia citricarpa* and the endophyte *Guignardia mangiferae*. *Plant Dis.* **2006**, *90*, 97–101.
55. Peres, N.A.; Harakava, R.; Carroll, G.C.; Adaskaveg, J.E.; Timmer, L.W. Comparison of molecular procedures for detection and identification of *Guignardia citricarpa* and *G. mangiferae*. *Plant Dis.* **2007**, *91*, 525–531.
56. van Gent-Peizer, M.P.E.; van Brouwershaven, I.R.; Kox, L.F.F.; Bonants, P.J.M. A Taqman PCR method for routine diagnosis of the quarantine fungus *Guignardia citricarpa* on citrus fruit. *J. Phytopathol.* **2007**, *155*, 357–363.
57. Schirmacher, A.M.; Tomlinson, J.A.; Barnes, A.V.; Barton, V.C. Species-specific real-timle PCR for diagnosis of *Phyllosticta citricarpa* on *citrus* species. *Bull. OEPP/EPPO Bull.* **2019**, *49*, 306–313.
58. Yang, Y.H.; Hu, J.H.; Chen, F.J.; Ding, D.K.; Zhou, C.Y. Development of a SCAR marker-based diagnostic method for the detection of the citrus target spot pathogen *Pseudofabraea citricarpa*. *Biomed. Res. Int.* **2018**, *2018*, 7128903.
59. Das, A.K.; Nerkar, S.; Gawande, N.; Thakre, N.; Kumar, A. Scar marker for phytophthora nicotianae and a multiplex PCR assay for simultaneous detection of *P. nicotianae* and *Candidatus* liberibacter asiaticus in citrus. *J. Appl. Microbiol.* **2019**, *127*, 1172–1183.

60. Pereira, W.V.; Bertolini, E.; Cambra, M.; Junior, N.S.M. Multiplex real-time PCR for detection and quantification of *Colletotrichum abscissum* and *C. gloeosporioides* on Citrus leaves. *Eur. J. Plant Pathol.* **2019**, *155*, 1–13.
61. Goh, T.K. Single-spore isolation using a hand-made glass needle. *Fungal Divers.* **1999**, *2*, 47–63.
62. Yin, L.F.; Chen, S.N.; Chen, G.K.; Schnabel, G.; Du, S.F.; Chen, C.; Li, G.Q.; Luo, C.X. Identification and characterization of three *Monilinia* species from plum in China. *Plant Dis.* **2015**, *99*, 1775–1783.
63. Farr, D.F.; Rossman, A.Y. *Fungal Databases*, 26 December 2018 ed.; U.S. National Fungus Collections, ARS, USDA: Washington DC, USA, 2018.
64. Chi, M.H.; Park, S.Y.; Lee, Y.H. A quick and safe method for fungal DNA extraction. *Plant Pathol. J.* **2009**, *25*, 108–111.
65. White, T.J.; Bruns, T.; Lee, S.; Taylor, J. Amplification and direct sequencing of fungal ribosomal RNA genes for phylogenetics. In *PCR Protocols: A Guide to Methods and Applications*; Innis, M.A., Gelfand, D.H., Sninsky, J.J., White, T.J., Eds.; Academic Press: San Diego, CA, USA, 1990; pp. 315–322.
66. Carbone, I.; Kohn, L.M. A method for desianing primer sets for speciation studies in filamentous ascomycetes. *Mycologia* **1999**, *91*, 553–556.
67. Glass, N.L.; Donaldson, G.C. Development of primer sets designed for use with the PCR to amplify conserved genes from filamentous ascomycetes. *Appl. Environ. Microb.* **1995**, *61*, 1323–1330.
68. Crous, P.W.; Groenewald, J.Z.; Risède, J.M.; Simoneau, P.; Hywel-Jones, N.L. *Calonectria* species and their *Cylindrocladium* anamorphs: Species with clavate vesicles. *Stud. Mycol.* **2004**, *50*, 415–430.
69. Hall, A.T. Bioedit: A user-friendly biological sequence alignment editor and analysis program for windows 95/98/nt. *Nucleic Acids Res.* **1999**, *41*, 95–98.
70. Swofford, D.L. *PAUP* Phylogenetic Analysis Using Parsimony, (*and Other Methods)*, Version 4.0 b10; Sinauer Associates: Sunderland, MA, USA, 2003.
71. Hillis, D.M.; Bull, J.J. An empirical test of bootstrapping as a method for assessing confidence in phylogenetic analysis. *Syst. Biol.* **1993**, *42*, 182–192.
72. Ronquist, F.; Teslenko, M.; Van der Mark, P.; Ayres, D.L.; Darling, A.; Höhna, S.; Larget, B.; Liu, L.; Suchard, M.A.; Huelsenbeck, J.P. Mrbayes 3.2: Efficient bayesian phylogenetic tnference and model choice across a large model space. *Syst. Biol.* **2012**, *61*, 539–542.
73. Nylander, J.A.A. *Mrmodeltest v.2. Program Distributed by the Author*; Evolitionary Biology Centre: Uppsala Univeristy: Uppsala, Sweden, 2004.
74. Rambaut, A. *Figtree v.1.4.2*; Institute of Evolutionary Biology, Ashworth Laboratories, University of Edinburgh:Edinburgh, UK, 2014.
75. Rayner, R.W. *A Mycological Colour Chart*; Commonwealth Mycological Institute and British Mycological Society: Kew, SurreyLondon, UK, 1970.
76. Lombard, L.; van Leeuwen, G.C.M.; Guarnaccia, V.; Polozzi, G.; van Rijswick, P.C.J.; Rosendahl, C.H.M.; Gabler, J.; Crous, P.W. *Diaporthe* species associated with *Vaccinium*, with specific reference to Europe. *Phytopathol. Mediterr.* **2014**, *53*, 287–299.
77. Aguilera-Cogley, V.; Vicent, A. Etiology and distribution of foliar fungal diseases of citrus in Panama. *Trop. Plant Pathol.* **2019**, *44*, 519–532.
78. Kanematsu, S.; Kobayashi, T.; Kudo, A.; Ohtsu, Y. Conidial morphology, pathogenicity and culture characteristics of *Phomopsis* isolates from peach, Japanese pear and apple in Japan. *Jpn. J. Phytopathol.* **1999**, *65*, 264–273.
79. Kanematsu, S. Phylogeny of phomopsis species from fruit trees. In *Direct Submission Sequence of Diaporthe citri Strian FCDC2*; National Institute of Fruit Tree Science, Apple Research Station: Morioka, Japan, 2007.
80. Gao, Y.H.; Su, Y.Y.; Sun, W.; Cai, L. *Diaporthe* species occurring on *Lithocarpus glabra* in China, with descriptions of five new species. *Fungal Biol.* **2015**, *119*, 295–309.
81. Mahadevakumar, S.; Yadav, V.; Tejaswini, G.S.; Sandeep, S.N.; Janardhana, G.R. First report of *Phomopsis citri* associated with dieback of *Citrus lemon* in India. *Plant Dis.* **2014**, *98*, 1281.
82. Polonio, J.C.; Almeida, T.T.; Garcia, D.; Mariucci, G.E.G.; Azevedo, J.L.; Rhoden, S.A.; Pamphile, J.A. Biotechnological prospecting of foliar endophytic fungi of guaco (*Mikania glomerata* Spreng.) with antibacterial and antagonistic activity against phytopathogens. *Genet. Mol. Res.* **2015**, *14*, 7297–7309.
83. Polonio, J.C.; Ribeiro, M.A.S.; Rhoden, S.A.; Sarragiotto, M.H.; Azevedo, J.L.; Pamphile, J.A. 3-nitropropionic acid production by the endophytic *Diaporthe citri*: Molecular taxonomy, chemical characterization, and quantification under ph variation. *Fungal Biol.* **2016**, *120*, 1600–1608.

84. Vasilyeva, L.N.; Rossman, A.Y.; Farr, D.F. New species of the Diaporthales from Eastern Asia and Eastern North America. *Mycologia* **2007**, *99*, 916–923.

© 2020 by the authors. Submitted for possible open access publication under the terms and conditions of the Creative Commons Attribution (CC BY) license (http://creativecommons.org/licenses/by/4.0/).
